# Levetiracetam for the treatment of mild cognitive impairment in Parkinson’s disease: a double-blind controlled proof-of-concept trial protocol

**DOI:** 10.1186/s40814-023-01406-y

**Published:** 2023-11-22

**Authors:** Nadeeka Dissanayaka, Dana Pourzinal, Gerard J. Byrne, Jihyun Yang, Katie L. McMahon, Gregory M. Pontone, John D. O’Sullivan, Robert Adam, Roberta Littleford, Mark Chatfield, Alexander Lehn, Zoltan Mari, Arnold Bakker

**Affiliations:** 1 UQ Centre for Clinical Research, The University of Queensland, Herston, QLD Australia; 2 Department of Neurology, Royal Brisbane & Women’s Hospital, Herston, QLD Australia; 3 School of Psychology, The University of Queensland, St Lucia, Brisbane, QLD Australia; 4 Mental Health Service, Royal Brisbane & Women’s Hospital, Herston, Brisbane, QLD Australia; 5 School of Clinical Sciences, Queensland University of Technology, Brisbane, QLD Australia; 6 Department of Neurology, Johns Hopkins University, Baltimore, USA; 7 Department of Psychiatry and Behavioral Sciences, Johns Hopkins University, Baltimore, USA; 8 Department of Neurology, Princess Alexandra Hospital, Woolloongabba, Brisbane, QLD Australia; 9 Cleveland Clinic Lou Ruvo Center for Brain Health, Las Vegas, USA

**Keywords:** Parkinson’s disease, Dementia, Levetiracetam, Episodic memory, fMRI, Hippocampus

## Abstract

**Background:**

Mild memory impairment, termed amnestic mild cognitive impairment (aMCI), is associated with rapid progression towards dementia in Parkinson’s disease (PD). Studies have shown hyperactivation of hippocampal DG/CA3 subfields during an episodic memory task as a biomarker of aMCI related to Alzheimer’s disease. This project investigates the feasibility of a trial to establish the efficacy of a repurposed antiepileptic drug, levetiracetam, in low doses as a putative treatment to target DG/CA3 hyperactivation and improve episodic memory deficits in aMCI in PD. Based on previous work, it is hypothesized that levetiracetam will normalize DG/CA3 overactivation in PD-aMCI participants and improve memory performance.

**Methods:**

Twenty-eight PD-aMCI participants, 28 PD participants without memory impairment (PD-nMI), and 28 healthy controls will be recruited. PD-aMCI participants will undertake a 12-week randomized, placebo-controlled, double-blind cross-over trial with a 14-day treatment of 125 mg levetiracetam or placebo twice daily, separated by a 4-week washout period. After each treatment period, participants will complete an episodic memory task designed to tax hippocampal subregion-specific function during high-resolution functional magnetic resonance imaging (fMRI). PD-nMI and healthy controls will undergo the fMRI protocol *only*, to compare baseline DG/CA3 subfield activity.

**Results:**

Episodic memory task performance and functional activation in the DG/CA3 subfield during the fMRI task will be primary outcome measures. Global cognition, PD severity, and adverse events will be measured as secondary outcomes. Recruitment, eligibility, and study completion rates will be explored as feasibility outcomes.

**Conclusions:**

This study, the first of its kind, will establish hippocampal subregion functional impairment and proof of concept of levetiracetam as an early therapeutic option to reduce dementia risk in PD.

**Trial registration:**

ClinicalTrials.gov, NCT04643327. Registered on 25 November 2020.

## Introduction

Memory impairment is pronounced in many people with mild cognitive impairment (MCI) in Parkinson’s disease (PD) [1] and is termed amnestic MCI (aMCI). Studies have repeatedly demonstrated that mild memory impairment in PD is associated with faster disease progression [2], more rapid cognitive decline [3–5], conversion to PD-MCI [6, 7], and an increased risk of subsequent dementia [4, 7–9]. To our knowledge, currently, there are no known interventions to reduce the risk of dementia in PD. This proof-of-concept study is a critical first step in identifying a neuroimaging biomarker of PD-aMCI and exploring the efficacy of the repurposed drug levetiracetam in improving episodic memory function and reducing dementia risk in PD.

Episodic memory is thought to critically depend on pattern completion and pattern separation. Pattern completion is the capacity to recall complete memories from partial cues, whereas pattern separation is the process of discriminating between highly similar experiences to encode them as non-overlapping representations [10]. While it is well established that structures of the medial temporal lobe are inextricably linked to memory function [11], recent studies have focused on the discrete role of hippocampal subregions in memory recall [12–14]. Specifically, the CA3 and dentate gyrus (DG) hippocampal subregions appear to play a key role in pattern separation and completion [10, 11, 15].

To determine the contribution of these hippocampal subregions to episodic memory function, Bakker et al. [12, 14] developed high-resolution functional magnetic resonance imaging (fMRI) methods in combination with a pattern separation task designed to tax subregion-specific function [16]. In several studies, Bakker and colleagues administered this fMRI paradigm to older adults with aMCI at risk of Alzheimer’s disease dementia [12, 14, 17]. Their results showed that aMCI participants exhibit a deficit in pattern separation and that this deficit was associated with hyperactivity localized to the DG/CA3 subregion of the hippocampus in aMCI participants compared to healthy controls. Bakker et al. [12, 14] also demonstrated that low doses of the antiepileptic medication levetiracetam resulted in a significant reduction in hippocampal DG/CA3 activity and was associated with improvement in pattern separation performance of aMCI participants [12, 14, 17]. These findings suggest that correcting hippocampal hyperactivation in with memory-dominant impairment in PD via low-dose levetiracetam can improve episodic memory function. Currently, the levetiracetam therapeutic approach is the subject of an FDA-registered NIH-supported phase III clinical trial (ClinicalTrials.org: *NCT03486938*) in the USA aimed to not only provide symptomatic relief but also as a disease-modifying approach in people with aMCI related to Alzheimer’s disease.

The present study will apply this approach to establish a proof of concept of the levetiracetam trial for aMCI in PD. The feasibility objectives of this proof-of-concept study aim to evaluate the viability of recruitment for the project and rates of eligibility, screen failure, and study completion to inform future trials. The first aim of the study is to confirm hippocampal DG/CA3 subregion dysfunction with fMRI during a task of episodic memory in PD-aMCI compared to people with PD without memory impairment (PD-nMI) and age-matched healthy controls. Based on a series of animal and human studies on hippocampal dysfunction in aging and memory impairment [14, 16, 18–20], it is hypothesized that PD-aMCI will exhibit increased activation within the hippocampal DG/CA3 subregion during the performance of an episodic memory task when compared to PD-nMI and healthy control participants, thereby providing a neuroimaging biomarker for therapeutic target development. The second aim of the study is to examine the repurposed use of low-dose levetiracetam (125 mg twice daily for 14 days) to reduce hippocampal DG/CA3 subfield hyperactivity and improve episodic memory function in PD-aMCI. Based on existing work in Alzheimer’s disease [12, 14, 16], low-dose levetiracetam treatment is expected to reduce the overactivation within the hippocampal DG/CA3 subregion in PD-aMCI and improve episodic memory performance that critically depends on this region, thereby providing a strong foundation for larger clinical studies of this repurposed drug to treat memory impairment in PD.

## Material and methods

### Trial design

**Fig. 1:**
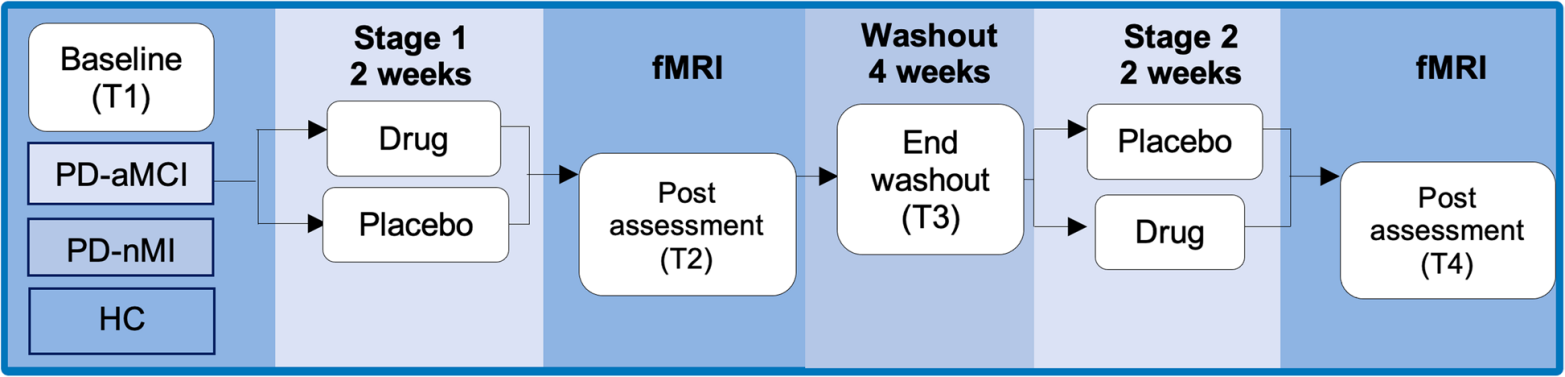
Study procedure flow chart. Legend: T = Time, PD = Parkinson’s disease, aMCI = amnestic mild cognitive impairment, nMI = no memory impairment, HC = healthy controls, fMRI = functional magnetic resonance imaging

Using the high-resolution fMRI pattern separation paradigm adopted from Bakker and colleagues [12], we will employ a randomized-controlled, double-blind, within-subject, crossover trial. Please refer to the study flow chart (Fig. 1) and the participant timeline (Table 1) for visual depictions of the design. All PD-aMCI participants will complete a baseline screening assessment, after which they will be randomized using block randomization at a 1:1 ratio into active (levetiracetam 125 mg twice daily [BID]) and placebo conditions. After 2 weeks on their respective treatment regimens (active or placebo), participants will be invited to undertake the first post-assessment where they will complete the fMRI pattern separation task. A 4-week washout period will follow, after which experimental conditions will crossover, and participants will begin their second treatment regimen. This will continue for 2 weeks and will be followed by a second post-assessment and fMRI task. Healthy controls and PD-nMI participants will only complete screening assessments and, if eligible, the fMRI task. The data collected from these groups provides a baseline against which hippocampal overactivation in PD-aMCI can be assessed.

**Table 1:** Participant timeline

Procedures	Screening		Study period				Close-out
	**Enrolment**	**Day 14** ***T***_**0**_***:* allocation**	**Day 0** ***T***_**1**_**: baseline**	**Day 14** ***T***_**2**_**: post-assessment 1**	**Day 44** ***T***_**3**_**: end washout period**	**Day 58** ***T4*: Post-assessment 2**	
Procedure for HC and PD-nMI groups							
Informed consent	X						
Screening interviews	X						
MRI session			X				
Procedure for PD-aMCI group							
Informed consent	X						
Screening interviews	X						
Bloodwork	X			X	X	X	
Medical assessment	X						
Random allocation		X					
Dispensing of medication		X			X		
Study commencement notification to treating physicians (GP/neurologist)		X					
Commence treatment (levetiracetam ↔ placebo)			X		X		
Post-treatment assessment				X		X	
MRI session				X		X	
Telephone check-in (weekly)			X	X	X	X	X
Report to GP/neurologist							X

*PD-aMCI* Parkinson’s disease with amnestic mild cognitive impairment, *PD-nMI* Parkinson’s disease without memory impairment, *HC* Healthy controls, *fMRI* functional magnetic resonance imaging

### Study setting and recruitment

Ethical approval has been received from the Royal Brisbane and Women’s Hospital (HREC/2020QRBW/69379) and the University of Queensland (20,200,002,745) Human Research Ethics Committees (HREC). Recruitment will take place in public hospitals and private neurology clinics, as well as in community settings such as local support groups and organizations. Consumer groups and support groups will be engaged to maximize advertisement effectiveness and assist with recruitment. An existing database of research participants who have consented to be contacted for future studies will also be used for recruitment. Eligible participants will be invited to complete questionnaires, interviews, medical assessments, and fMRI scans. Blood collection and analysis will take place at the hospital pathology clinic and drug dispensing will take place at the hospital pharmacy.

### Eligibility criteria

Eligibility will be assessed during the screening interviews administered by study investigators and, additionally for PD-aMCI participants, through a medical assessment performed by the study doctor. Inclusion criteria comprise persons with PD and age- and gender-matched healthy older adults who are fluent in English. Healthy controls and PD-nMI participants will be excluded if they show (1) dementia as defined by a score below 19 on the Montreal Cognitive Assessment (MoCA); (2) contraindication to having MRI; (3) bipolar disorder, schizophrenia, alcohol, or substance use disorders; (4) current diagnosis of major depression or current suicidal ideation as measured by the UQ Psychology Suicide Risk Assessment; (5) difficulty complying with protocol requirements; (6) significant non-PD neurological disease; or (7) other clinically significant disease. PD-aMCI participants will also be excluded if they demonstrate: (1) signs of vascular dementia as measured by the Modified Hachinski Scale; (2) history of seizures or sensitivity to levetiracetam; (3) use of anticonvulsant medication within 3 months of screening; (4) use of other excluded medications (antihistamines with anticholinergic properties, opiates); (5) severe renal impairment; or (6) clinically significant abnormalities on B12 or thyroid function test.

### Informed consent

In adherence with Australian National Health and Medical Research Council (NHMRC) guidelines, a participant information sheet will be provided and written informed consent will be obtained from all participants prior to study commencement. If participants show interest in participating, they will be asked to sign a consent form prior to their first interview.

### Intervention

Levetiracetam is a medication currently approved in Australia for the management of epilepsy. While its exact mechanism of action is not fully known, levetiracetam is reported to modulate the release of synaptic neurotransmitters by binding to the synaptic vesicle protein SV2A, thereby reducing neuronal excitability and the likelihood of seizures [21]. Previous trials have also investigated levetiracetam for the treatment of dyskinesia in PD, reporting adverse events including worsening of PD symptoms, fatigue, and somnolence [22, 23]. However, effective doses of levetiracetam used in Alzheimer’s disease-related MCI are much lower than those reported in previous PD trials and are required clinically for antiepileptic efficacy (1000–3000 mg/day). Bakker et al. [12] found that low-dose levetiracetam at 62.5 mg BID and 125 mg BID, but not 250 mg BID, attenuated hippocampal overactivity and improved task-related memory function [12]. The 125 mg dose was therefore applied in the present study, with the exclusion of people with a history of sensitivity to levetiracetam.

Both investigational drug and placebo conditions will be administered in oral capsule form. A pharmaceutical manufacturer will produce and label active and matching placebo capsules. The placebo as well as the backfilling for the levetiracetam capsules will be compounded from a mixture of maize starch and pre-gelatinized maize starch. For each condition, 36 capsules will be distributed to the participant by the hospital pharmacy to sufficiently supply for 14 days of treatment plus an additional four days in case of scheduling conflicts. Capsules will be provided to participants in Websterpaks® labeled with appropriate instructions. Participants will be instructed to take the capsules twice daily, once in the morning and once again in the evening, with their final capsule taken on the morning of their MRI assessment.

Due to the low doses of levetiracetam to be administered in this study, there will be no option to reduce the dose for trial participants. Discontinuation can occur voluntarily at the request of the participant or at the discretion of the study doctor or primary care physician. Noncompliance defined as taking < 80% or > 120% of the study drug during any evaluation period will result in discontinuation. Potential methods for increasing intervention compliance will be discussed with participants (e.g., pill timer, liaising with a support person, diary, following Websterpak® labels). Drug compliance will be measured by both checking returned study drug containers and taking blood samples at the end of each treatment phase to measure serum levetiracetam levels.

All concomitant medication will be recorded throughout the study period. To preserve optimal PD treatment, changes to PD medication will be permitted, with all changes recorded during the trial period. Most concomitant care methods are permitted during the trial for PD-aMCI participants except those outlined in the exclusion criteria such as anticonvulsant/antiepileptic medications. Concomitant experimental interventions are not permitted.

### Outcomes

The primary outcomes of the present study are change in mean episodic memory function as measured by the pattern separation task and DG/CA3 hippocampal subfield functional activity during the pattern separation task measured using fMRI [16], with both outcomes measured at the end of each treatment phase. These outcomes were chosen due to their relevance to the aMCI subtype [12, 14, 17] and so that the potential functional mechanisms underlying episodic memory impairment in PD-aMCI could be both evaluated and targeted. Change in episodic memory, global cognitive ability, and parkinsonism from baseline will also be measured at the end of each treatment phase using the Buschke Selective Reminding Test [24], MoCA [25], and Movement Disorder Society Unified Parkinson’s Disease Rating Scale (MDS-UPDRS) [26], respectively. Alternative versions of the Buschke and MoCA will be used to reduce practice effects. Finally, feasibility outcomes of recruitment, eligibility, and study completion rates will be explored to determine the feasibility of a larger phase II trial.

### Sample size

Because of inherited limitations in designing power calculations for fMRI studies [27, 28], the sample size estimate was first calculated using the pre-post change of episodic memory function on the pattern separation task observed in the previous levetiracetam trials in elderly adults with aMCI [12]. It was therefore estimated that a sample size of 24 PD-aMCI participants would provide 80% power to detect a difference of 0.07 between levetiracetam and placebo-treated conditions using a paired *t* test assuming a standard deviation of the difference between levetiracetam and placebo condition is 0.12 and 5% significance level. A sample size estimate for the hippocampal activation analyses was also calculated, based on the previously reported effect sizes of DG/CA3 activity observed during the critical trials of the pattern separation task between aMCI and healthy control groups (*d* = 0.94, *d* = 0.91, *d* = 1.12) [12]. A sample size of 24 participants per group will provide > 80% power to detect differences in activity between groups of size *d* = 0.9 and is consistent with attempts to quantify optimal sample sizes in fMRI studies that treat participants as random effects [27, 28]. Based on previous work in PD, we will account for 14% attrition and target 28 participants per group.

### Allocation and blinding

The order of treatment/placebo conditions will be decided using a computer-generated random selection software set up by an independent biostatistician to allocate participants into active-first or placebo-first groups at a 1:1 ratio. The allocation list will be handled by the hospital pharmacy, who will randomly allocate participants into the active-first or placebo-first groups using the computer-generated sequence. Trial participants will be blinded to the experimental conditions by being administered physically identical pills for each condition. All assessors (interview, fMRI measures), care providers, and data analysts will also be blinded to the experimental conditions. This will be achieved by delegating all allocation responsibilities to the hospital pharmacy. Unblinding will be permissible if a suspected unexpected serious adverse reaction occurs. In this case, the research assistant will be responsible for accessing the allocation information and relaying the relevant participant ID and experimental condition to the appropriate party.

### Data collection

Demographic and covariate data will be collected via an online questionnaire, two screening interviews, and a medical assessment. The primary and secondary outcome variables will be measured in the post-treatment interview and each MRI session. Adverse events will be recorded on a weekly basis either in person at each assessment or via a brief telephone call with the participant. Comprehensive training will be provided to study investigators responsible for data collection and REDcap data entry.

#### Screening measures

Preliminary screening information will be collected via an online questionnaire, collecting demographic, and PD-related information. The Geriatric Anxiety Inventory (GAI) [29] and Geriatric Depression Scale-15 (GDS-15) [30] will also be administered in the questionnaire, which are highly validated measures of anxiety and depression for older adults [31, 32]. The Parkinson’s Anxiety Scale (PAS) [33] and Starkstein Apathy Scale (SAS) [34] will also be administered to PD participants to measure PD-specific symptoms of anxiety and apathy. To assess functional impairment, a self-report version of the widely implemented Lawton Instrumental Activities of Daily Living (IADL) [35] will be administered to all participants. Two questions assessing subjective memory complaints will be included, both of which have been validated in a large-scale sample of older, Australian adults [36].

Participants will also undertake two screening interviews. The first interview will include the MoCA as a validated measure of global cognition [25], the MDS-UPDRS as a validated measure of parkinsonism [26], and the Buschke Selective Reminding Test [24] as a measure of episodic memory ability, which has been validated in populations related to PD-aMCI including neurological disease and Alzheimer’s disease samples [37]. To identify dementia, participants will be administered the Clinical Dementia Rating Scale (CDRS), which has been highly validated in older populations [38]. The MINI-plus [39] will also be administered to identify any psychological conditions.

The second interview is a cognitive test battery adhering to guidelines by the *Movement Disorder Society (MDS)* for identifying PD-MCI [40]. The battery comprises 10 tests; two within each of the five cognitive domains (memory, attention, executive, visuospatial, language). Each of the selected tests was recommended by the *MDS* and has been validated in PD. The battery comprises the Trail Making Test (TMT) [41], STROOP color-word-sorting test [42], card-sorting test (Delis-Kaplan Executive Function System) [43], verbal fluency (Delis-Kaplan Executive Function System) [43], Hopkins Verbal Learning Test-Revised (HVLT-R) [44], Brief Visuospatial Memory Test-Revised (BVMT-R) [44], Benton’s Judgement of Line Orientation [45], CLOX clock-drawing task [46], semantic fluency (Delis-Kaplan Executive Function System) [43], and the Boston Naming Test (BNT) [47]. Scores 1.5 standard deviations (*SD*) below normative values indicate a failed test, with two or more failed tests fulfilling criteria for PD-MCI provided that functional ability is preserved. In line with *MDS* recommendations [40], the Lawton Instrumental Activities of Daily Living Scale (IADL) will be used to measure functional ability. Memory impairment will be defined by failure of any of the three memory tests (i.e., HVLT-R, BVMT-R, or Buschke). Those with demonstrated memory impairment will be considered PD-aMCI, and those with no memory impairment will be considered PD-nMI. Consultant psychiatrists and neurologists on the investigatory team will supervise and advise on the assessment procedure and diagnosis of PD-aMCI.

Finally, a medical assessment performed by the study doctor will evaluate PD-aMCI participants’ capacity to partake in the clinical trial. This assessment will evaluate the results of the B12, thyroid, creatinine, liver function, and full blood count tests. Baseline symptoms will be established to serve as a comparison for future adverse effects to evaluate their relationship to the study drug. The Modified Hachinski Ischemic Scale [48], a widely used scale for vascular dementia, will also be administered. The study doctor will also perform a physical neurological examination and check the participants’ medical history.

#### MRI session

**Fig. 2:**
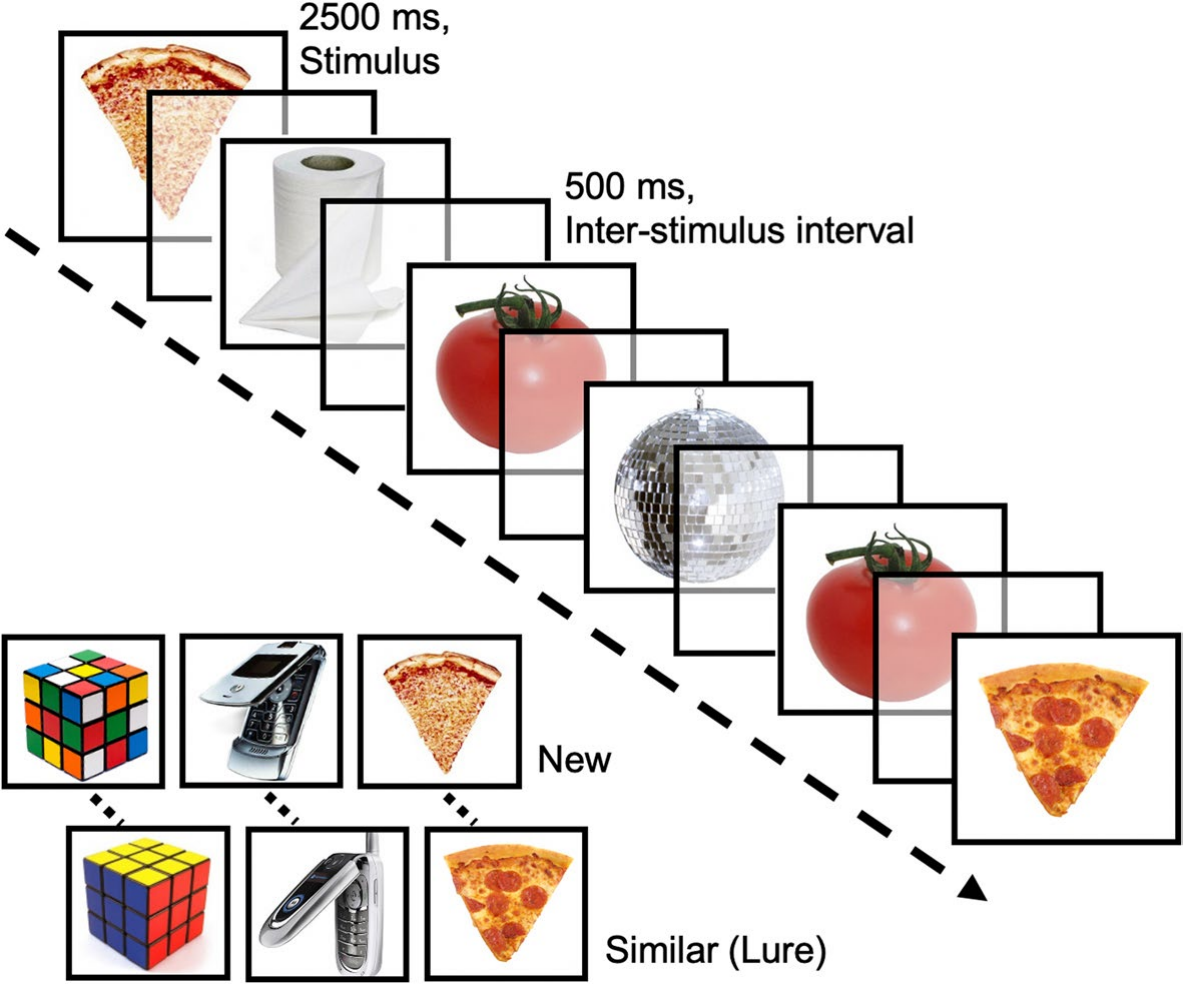
Pattern separation task. Legend: Participants are shown a series of pictures of everyday objects and asked to determine if the item is “new” in the context of the experiment, “old” (exact repetition of a previously shown item), or “similar” but not exactly the same, termed “lure” trials. Correct identification of the critical lure trials is thought to depend on hippocampal subregion dentate gyrus (DG)/CA3-mediated functions shown to be associated with age-related memory impairment and memory impairment in amnestic mild cognitive impairment [12]

All participants will undergo MRI scanning. PD-nMI and healthy controls will have one scan after the final screening interview. PD-aMCI participants will be scanned twice, once at the end of each treatment phase. These sessions will collect data for the primary outcome variables, being functional brain activity and behavioral performance during a pattern separation paradigm (Fig. 2). In this paradigm, participants are presented with 768 pictures of common, everyday objects. The series comprises 384 items that are new in the context of the task, 96 items that are an exact repetition of a previous item (i.e., old) and 96 items that are similar but not exactly the same termed “lures.” Participants are tasked with determining whether the item is “new,” “old,” or “similar.” An item is correctly judged “new” if it is seen for the first time in the context of the task, and “old” if the item is repeated. The third option of “similar” is the correct judgment only when an object resembles an item previously seen in the task. These “similar lures” are the critical trials for the assessment of DG/CA3 contribution to memory performance, since prior work has shown that correct identification of “similar” items depends on DG/CA3 mediated pattern separation [16].

#### Post-treatment

For PD-aMCI participants who are enrolled in the clinical trial, brief medical assessments will be conducted at T2, T3, and T4 to record adverse effects and provide the opportunity for the participant to ask any questions. At T2, T3, and T4, PD-aMCI participants will also provide blood samples for measuring of levetiracetam blood plasma concentration and full blood count. Blood plasma levels of levetiracetam will be measured to record the actual dose.

### Data management and confidentiality

Information related to the study will be stored on the secure Research Data Manager (RDM) server of The University of Queensland, and participant data will be stored on the secure REDcap server. Access to these servers will be granted to a minimal number of study investigators for data maintenance and quality control. The RDM will be backed up to an internal centralized research data drive. Hard copies of the study data will be securely stored in locked cabinets at UQCCR. Data entry will be completed by a study team member as assessments are completed. All data will be coded and depersonalized using participant identification numbers at the point of data entry. Confidentiality will be preserved when transferring data by ensuring that no personally identifiable information is transferred from the central database. In other circumstances (e.g., presentations) privacy will be protected by de-identifying the participant. At the end of the study, all study-related data and files will be archived for 15 years to comply with Good Clinical Practice (GCP) guidelines, after which the information will be destroyed securely.

### Statistical methods

fMRI data and the pattern separation task performance will be analyzed using methods described previously in detail [12–14]. Briefly, the fMRI data will undergo standard pre-processing steps, behavioral vectors will be created identifying each of the task trial types, and a deconvolution approach will be employed to assess activation in a given voxel for each task condition. Using the healthy control data, an ANOVA of the task condition will be used to select voxels that show task-related activation within the DG/CA3 hippocampal subregion. Within this voxel cluster the mean activation during the critical “lure” trials correctly identified as “similar” will be calculated for each participant. Missing data will be handled by using complete case analysis.

To test the hypothesis that within the cluster of task-related activation localized to the DG/CA3 subregion of the hippocampus, participants with PD-aMCI on placebo show increased activation when compared to PD-nMI and healthy controls, a repeated measures ANOVA with planned contrasts will be used. The same approach will be used to test whether participants with PD-aMCI on placebo correctly identify “lure” items less often compared to PD-nMI and healthy controls. To test the hypothesis that levetiracetam treatment reduces hippocampal hyperactivation, a paired-samples *t* test will be employed to compare mean activation during critical “lure” trials in the DG/CA3 in the active versus placebo PD-aMCI conditions. A paired-samples *t* test will also be used to compare the proportion of correctly identified “lure” trials in the active versus placebo PD-aMCI conditions.

### Reporting harms

Adverse events will be defined as any untoward medical event experienced by a participant throughout the trial. Medical events which may jeopardize the participant or require intervention to prevent death, hospitalization, significant disability/incapacity, or any other immediately life-threatening situation will be recorded as a serious adverse event and reported to the HREC as soon as possible. Abnormal laboratory findings observed during the study period that are judged by the study doctor to be clinically significant adverse events will also be recorded. The study doctor will use clinical judgment to determine the relationship between the investigational product and the occurrence of each adverse event, considering both the temporal relationship of the event to the product and potential alternative causes such as the history of underlying diseases or concomitant therapies. Post-study adverse events (events that occur after close-out) will not be sought out, but in the case that an investigator learns of any serious adverse event at any time after participants’ involvement in the study, and they consider the event to be related to the investigational product, the investigator will report the adverse event to the study sponsor as soon as possible.

## Discussion

Memory problems impair the quality of life of individuals with PD [49]. In particular, the hefty disease burden of dementia [50] has motivated research in pursuit of a therapy to treat or delay the rapid cognitive decline frequently associated with PD-MCI [8, 51]. The current proof-of-concept protocol proposes the repurposing of an antiepileptic medication, levetiracetam, to treat mild memory impairment and reduce the risk of dementia in PD. Parallel to this, a neuroimaging biomarker for memory impairment in PD will be evaluated to serve as a novel therapeutic target. The feasibility of the proof-of-concept trial will be explored in depth to justify the continuation of the study as a larger, multi-site phase II trial.

There are several strengths to the proposed protocol. Firstly, the project is a collaborative effort, bringing together an assembly of multidisciplinary experts from Australia and the USA to create a highly experienced study team. Our Consumer and Community Involvement Group (CCIG) comprised of people with lived experience of Parkinson’s disease, mild cognitive impairment, and dementia were also consulted during the study design process to ensure that the study is adequately designed to meet the needs of the participants who will be targeted. Repurposing an approved drug is also a great strength, as this will bypass several phases of the typical drug development process and shorten the potential research translation timeline from evidence to practice. This also means that the safety profile of the investigational drug is readily accessible. The safety profile of levetiracetam in PD indicates a safe dose of up to 3000 mg/day [52, 53], which is much higher than the investigated dose in this trial (250 mg/day). Additionally, as participants will primarily be recruited from an existing database, this streamlines the recruitment process and allows prior identification of participants who are likely eligible for the study (e.g., previously had MRI [54] and exhibited memory impairments in previous cognitive testing [55]).

Certain considerations must also be made. As the investigated demographic will be people with PD, it is important to prepare for movement within the MRI scanner due to the disruption this can cause to the fMRI data collection. While there may be data loss due to head movement, the investigators will ensure that this is minimalized (e.g., using head padding in the scanner and correcting for minor head movements post hoc). The project will also require certain concessions to meet the needs of the older demographic. As such, participants will be advised that they may take breaks during the assessments and, if requested, may be escorted by a study investigator to pathology. Fortunately, clinical rooms and the scanner are located on hospital premises, reducing the travel requirements of participants as they move between institutes.

### Trial status

Protocol version 5.0, Nov 2021. Recruitment commenced in February 2021. The expected completion date is Nov 2024.

## Data Availability

The datasets generated during the current study are not publicly available but may be available from the corresponding author on reasonable request.

## Abbreviations

AE: Adverse event
BNT: Boston Naming Test
BVMT-R: Brief Visuospatial Memory Test
CA: Cornu ammonus
CDRS: Clinical Dementia Rating Scale
DG: Dentate gyrus
DKEFS: Delis Kaplan Executive Function Scale
DSM: Diagnostic Statistical Manual
fMRI: Functional magnetic resonance imaging
GAI: Geriatric Anxiety Inventory
GCP: Good Clinical Practice
GDS: Geriatric Depression Scale
HC: Healthy control
HIRF: Herston Imaging Research Facility
HIS: Haschinski Ischemic Scale
HVLT: Hopkin’s Verbal Learning Test
IADL: Instrumental Activities of Daily Living
MCI: Mild cognitive impairment
MDS-UPDRS: Movement Disorder Society Unified Parkinson’s Rating Scale
MINI: Mini International Neuropsychiatric Interview
MoCA: Montreal Cognitive Assessment
NHMRC: National Health and Medical Research Council
PD: Parkinson’s disease
PD-aMCI: Parkinson’s disease with amnestic mild cognitive impairment
PD-nMI: Parkinson’s disease with no memory impairment
RDM: Research data manager
TMT: Trail Making Test
UQCCR: University of Queensland Centre for Clinical Research
